# Author Correction: The geology and evolution of the Near-Earth binary asteroid system (65803) Didymos

**DOI:** 10.1038/s41467-024-54189-y

**Published:** 2024-11-15

**Authors:** Olivier Barnouin, Ronald-Louis Ballouz, Simone Marchi, Jean-Baptiste Vincent, Harrison Agrusa, Yun Zhang, Carolyn M. Ernst, Maurizio Pajola, Filippo Tusberti, Alice Lucchetti, R. Terik Daly, Eric Palmer, Kevin J. Walsh, Patrick Michel, Jessica M. Sunshine, Juan L. Rizos, Tony L. Farnham, Derek C. Richardson, Laura M. Parro, Naomi Murdoch, Colas Q. Robin, Masatoshi Hirabayashi, Tomas Kahout, Erik Asphaug, Sabina D. Raducan, Martin Jutzi, Fabio Ferrari, Pedro Henrique Aragao Hasselmann, Adriano CampoBagatin, Nancy L. Chabot, Jian-Yang Li, Andrew F. Cheng, Michael C. Nolan, Angela M. Stickle, Ozgur Karatekin, Elisabetta Dotto, Vincenzo Della Corte, Elena Mazzotta Epifani, Alessandro Rossi, Igor Gai, Jasinghege Don Prasanna Deshapriya, Ivano Bertini, Angelo Zinzi, Josep M. Trigo-Rodriguez, Joel Beccarelli, Stavro Lambrov Ivanovski, John Robert Brucato, Giovanni Poggiali, Giovanni Zanotti, Marilena Amoroso, Andrea Capannolo, Gabriele Cremonese, Massimo Dall’Ora, Simone Ieva, Gabriele Impresario, Michèle Lavagn, Dario Modenini, Pasquale Palumbo, Davide Perna, Simone Pirrotta, Paolo Tortora, Marco Zannoni, Andrew S. Rivkin

**Affiliations:** 1 Johns Hopkins University Applied Physics Laboratory, Laurel, MD USA; 2 Southwest Research Institute, Boulder, CO USA; 3 DLR Institute of Planetary Research, Berlin, Germany; 4 University of the Côte d’Azur, Observatory of the Côte d’Azur, CNRS, Laboratory Lagrange, Nice, France; 5 University of Maryland, College Park, MD USA; 6 University of Michigan, Ann Arbor, MI USA; 7 INAF-Astronomical Observatory of Padova, Padova, Italy; 8 Planetary Science Institute, Tucson, AZ USA; 9 The University of Tokyo, Department of Systems Innovation, School of Engineering, Tokyo, Japan; 10 Institute of Astrophysics of Andalusia, CSIC, Granada, Spain; 11 University of Alicante, Alicante, Spain; 12 Superior Institute of Aeronautics and Space, University of Toulouse, Toulouse, France; 13 Daniel Guggenheim School of Aerospace Engineering, Georgia Institute of Technology, Atlanta, GA USA; 14 Univ. of Helsinki, Helsinki, Finland; 15 Lunar and Planetary Laboratory, University of Arizona, Tuscon, AZ USA; 16 Univ. of Bern, Bern, Switzerland; 17 Politechnic University of Milan, Milan, Italy; 18 INAF- Astronomical Observatory of Rome, Rome, Italy; 19 Royal Observatory of Belgium, Brussels, Belgium; 20 INAF-Institue of Space Astrophysics and Planetology, Rome, Italy; 21 Institue of Applied Physics “Nello Carrara”, CNR, Florence, Italy; 22 University of Bologna, Bologna, Italy; 23 University of Parthenope, Parthenope, Italy; 24 ASI, Rome, Italy; 25 Institute of Space Sciences (CSIC-IEEC), Barcelona, Catalonia Spain; 26 Univ. of Padova, Padova, Italy; 27 INAF- Astronomical Observatory of Trieste, Trieste, Italy; 28 INAF- Astronomical Observatory of Arcetri, Arcetri, Italy; 29 INAF- Astronomical Observatory of Capodimonte, Capodimonte, Italy

**Keywords:** Asteroids, comets and Kuiper belt, Geomorphology

Correction to: *Nature Communications* 10.1038/s41467-024-50146-x, published online 30 July 2024

In this article the funding from the Spanish project PID2021-128062NB-I00 funded by MCIN/AEI was omitted. The original article has been corrected.

