# Supplementary material for: The geology and evolution of the Near-Earth binary asteroid system (65803) Didymos
Source: Nat Commun. 2024 Jul 30;15:6202. doi: 10.1038/s41467-024-50146-x (PMC11289119; doi:10.1038/s41467-024-50146-x)
Supplement: Supplementary file 1 — Supplementary Information [file 41467_2024_50146_MOESM1_ESM.pdf]

Supplemental Material: The geology and evolution of the Near-Earth binary asteroid system (65803) Didymos.

Olivier Barnouin[1,*], Ronald-Louis Ballouz[1], Simone Marchi[2], Jean-Baptiste Vincent[3], Harrison Agrusa[4,5], Yun Zhang[6], Carolyn M. Ernst[1], Maurizio Pajola[7], Filippo Tusberti[7], Alice Lucchetti[7], R. Terik Daly[1], Eric Palmer[8], Kevin J. Walsh[2], Patrick Michel[4,9], Jessica M. Sunshine[5], Juan L. Rizos[10], Tony L. Farnham[5], Derek C. Richardson[5], Laura M. Parro[11], Naomi Murdoch[12], Colas Q. Robin[12], Masatoshi Hirabayashi[13], Tomas Kahout[14], Erik Asphaug[15], Sabina D. Raducan[16], Martin Jutzi[16], Fabio Ferrari[17], Pedro Henrique Aragao Hasselmann[18], Adriano Campo Bagatin[11], Nancy L. Chabot[1], Jian-Yang Li[8], Andrew Cheng[1], Michael C. Nolan[15], Angela M. Stickle[1], Ozgur Karatekin[19], Elisabetta Dotto[18], Vincenzo Della Corte[20], Elena Mazzotta Epifani[18], Alessandro Rossi[21], Igor Gai[22], Jasinghege Don Prasanna Deshapriya[18], Ivano Bertini[23], Angelo Zinzi[24], Josep M. Trigo-Rodriguez[25], Joel Beccarelli[26], Stavro Lambrov Ivanovski[27], John Robert Brucato[28], Giovanni Poggiali[28], Giovanni Zanotti[16], Marilena Amoroso[24], Andrea Capannolo[17], Gabriele Cremonese[7], Massimo Dall'Ora[29], Simone Ieva[18], Gabriele Impresario[24], Michèle Lavagn[17], Dario Modenini[22], Pasquale Palumbo[23], Davide Perna[18], Simone Pirrotta[24], Paolo Tortora[22], Marco Zannoni[22], and Andrew S. Rivkin[1]

[1]Johns Hopkins University Applied Physics Laboratory, Laurel, MD (olivier.barnouin@jhuapl.edu)
[2]Southwest Research Institute, Boulder, CO
[3]DLR Institute of Planetary Research, Berlin, Germany
[4]University of the Côte d'Azur, Observatory of the Côte d'Azur, CNRS, Laboratory Lagrange, Nice, France
[5]University of Maryland, College Park, MD
[6]University of Michigan, Ann Arbor, MI
[7]INAF-Astronomical Observatory of Padova, Padova, Italy
[8]Planetary Science Institute, Tucson, AZ
[9]The University of Tokyo, Department of Systems Innovation, School of Engineering, Tokyo, Japan
[10] Institute of Astrophysics of Andalusia, CSIC, Granada, Spain
[11]University of Alicante, Alicante, Spain
[12]Superior Institute of Aeronautics and Space, University of Toulouse, Toulouse, France
[13]Daniel Guggenheim School of Aerospace Engineering, Georgia Institute of Technology, Atlanta, GA
[14]Univ. of Helsinki, Helsinki, Finland
[15]Lunar and Planetary Laboratory, University of Arizona, Tuscon, AZ
[16]Univ. of Bern, Bern, Switzerland

[17]Politechnic University of Milan, Milan, Italy
[18]INAF- Astronomical Observatory of Rome, Rome, Italy
[19]Royal Observatory of Belgium, Brussels, Belgium
[20]INAF-Institue of Space Astrophysics and Planetology, Rome, Italy
[21]Institue of Applied Physics “Nello Carrara”, CNR, Florence, Italy
[22]University of Bologna, Bologna, Italy
[23]University of Parthenope, Parthenope, Italy
[24]ASI, Rome, Italy
[25]Institute of Space Sciences (CSIC-IEEC), Barcelona, Catalonia, Spain
[26]Univ. of Padova, Padova, Italy
[27]INAF- Astronomical Observatory of Trieste, Trieste, Italy
[28]INAF- Astronomical Observatory of Arcetri, Arcetri, Italy
[29]INAF- Astronomical Observatory of Capodimonte, Capodimonte, Italy

*Corresponding author: Olivier Barnouin, olivier.barnouin@jhuapl.edu

This supplementary materials section possesses the figures and table referenced in the Method section of the main paper. It also has a section on the unpublished geometric diameter-depth measurements for Bennu, that were used as comparison with the craters measured on Dimorphos. It provides the data used to make Main Fig. 7. All the other data are available in the PDS.

Supplementary Figures

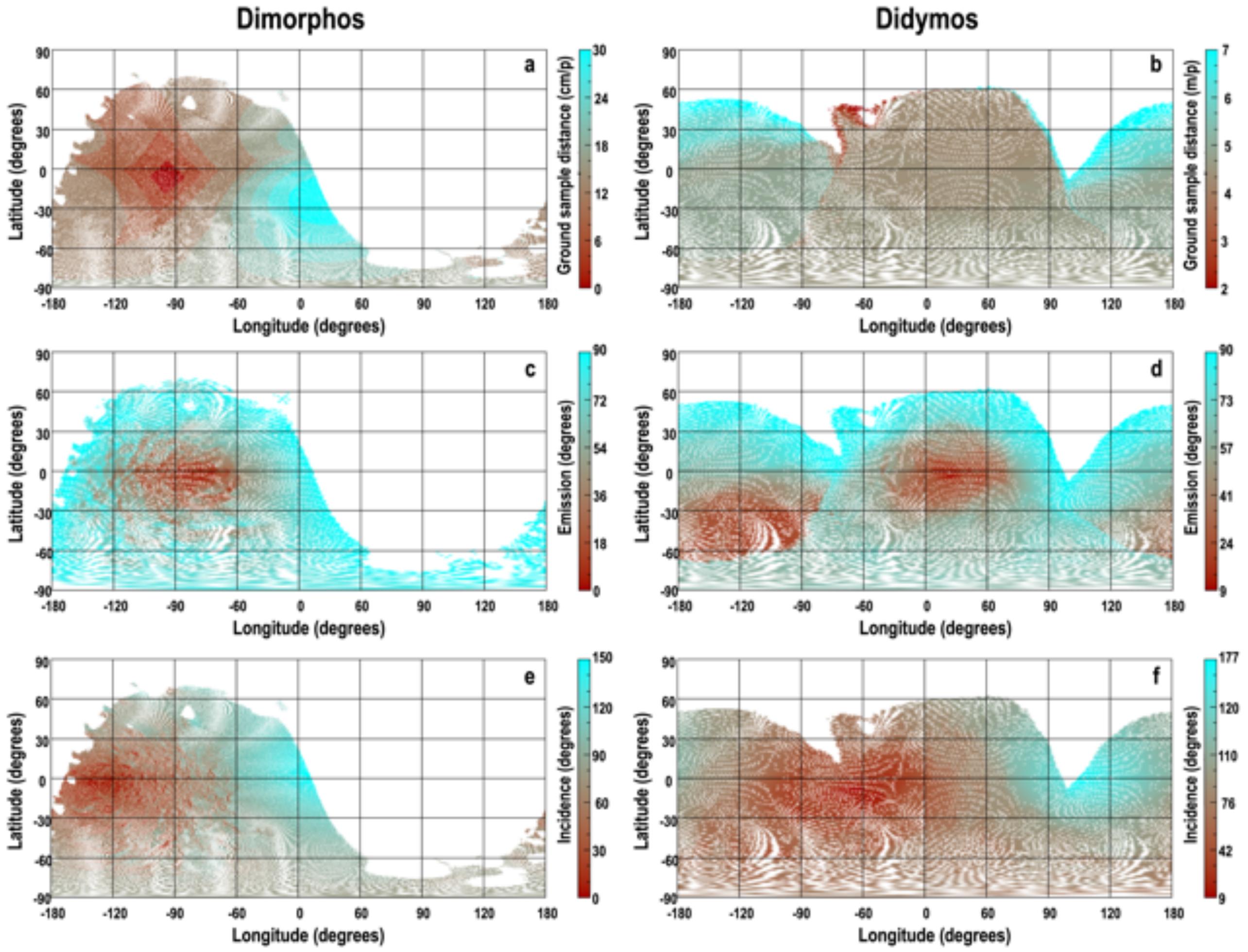

***Supplementary Figure 1**. Ground sample distance (a,b), emission (c,d) and incidence (e,f) coverage of DRACO data collected at Dimorphos (left-column:a,c,e), and combined DRACO and LUKE data collected at Didymos (right-column:b,d,f).*

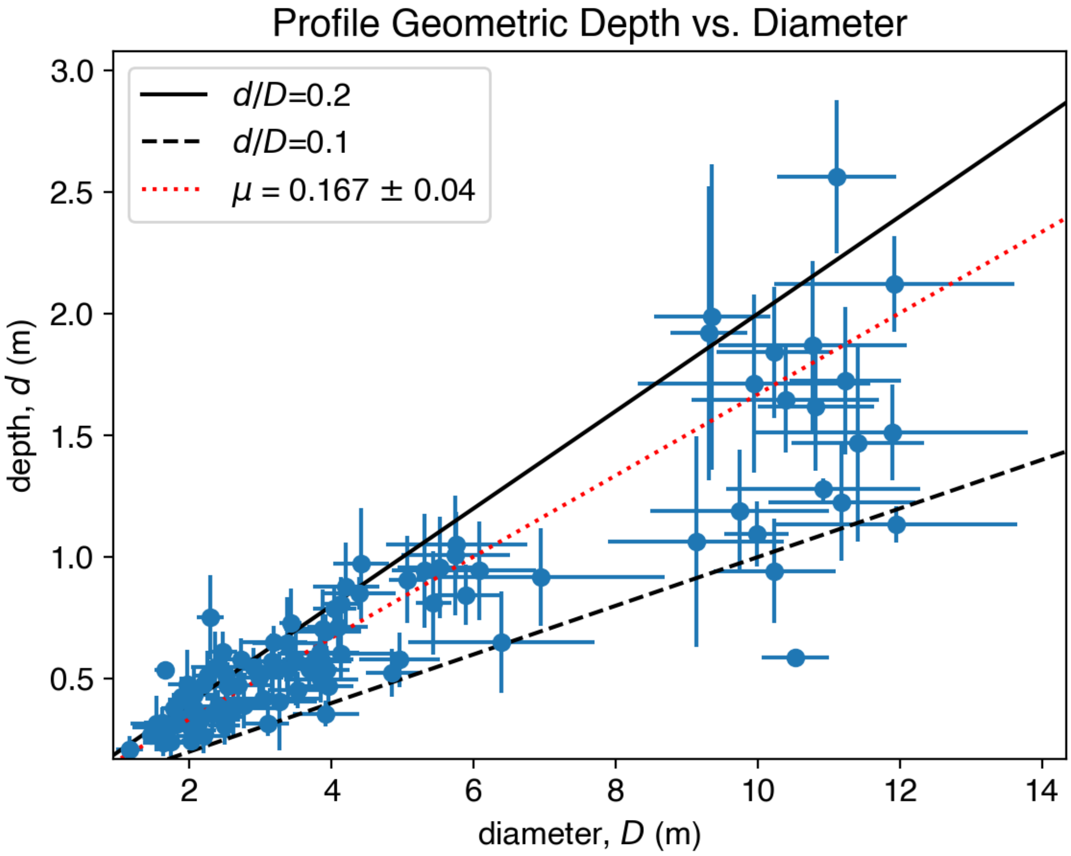

Supplementary Figure 2*. The geometric shapes of craters on Bennu with D< 12m. They compare favorably to the geometric shapes of all craters measured on Dimorphos, the largest of which has a D~11.1m . These crater shapes were collected while preparing Daly et al. (2020)[28] but were not published before because measurements that use surface elevation (which include the effects of gravity) are more useful for understanding and comparing the geological processes responsible for crater formation and modification on asteroids and planets, especially given recent findings that asteroid surface are very weak (see main manuscript for more details). Surface elevation was not computed for Dimorphos because of the challenges the proximity of Didymos poses to define a reasonable reference geoid. The Bennu data used to make this figure are available in the source data file of the main article.*

Tables

Supplementary Table 1. Didymos Global Digital Terrain Model

Parameter	Value
Volume (km^3)	0.203 $\pm$ 3.4%
Surface area (km^2)	1.729732$\pm$ 3.3%
Extent	
x (m)	819 $\pm$ 14
y (m)	801 $\pm$ 14
z (m)	607 $\pm$ 14
Best fit ellipsoid semi-major axes	
a (m)	409 $\pm$ 7
b (m)	398 $\pm$ 7
c (m)	295 $\pm$ 7
Equivalent radius (m)	365 $\pm$ 4
Density (kg/m^3)	2800 $\pm$ 280
Average slope (degrees)	34.0 $\pm$ 15.0
Median slope (degrees)	33

Supplementary Table 2. Crater candidate confidence criterion

Confidence Rating	Criteria
1	Circular uplifted rim, bowl shape
2	Partial circular rim; accumulation of boulders, some bowl shaped appearance, crushed interior
3	Circular structure, central depression present
4	Circular flat facet*

Supplementary Table 3. Candidate craters identified on Didymos.

Crater Candidate	Image	Location	Diameter (km)	Confidence
Dcrt01	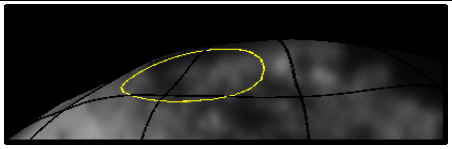	39.6N 2.8E	0.167	2
Dcrt02	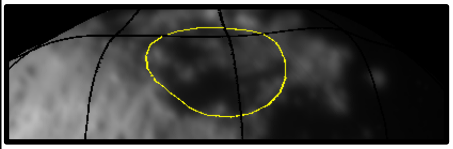	20.6N 25.9E	0.198	2
Dcrt03	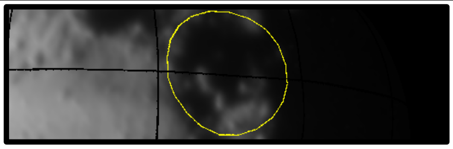	1.0N 44.5E	0.188	2
Dcrt04	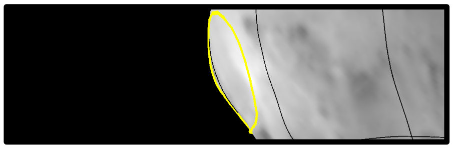	16.6S 309.1E	0.154	3
Dcrt05	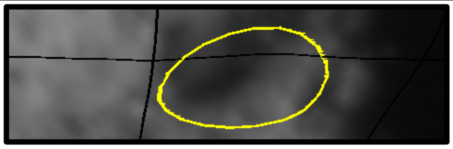	33.6S 40.7E	0.109	2
Dcrt06	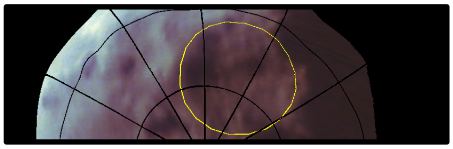	53.6S 21.9E	0.268	3
Dcrt07	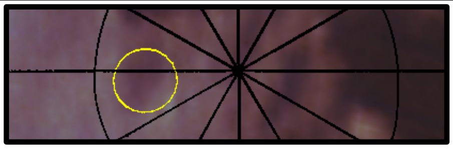	70.40S 263.8E	0.072	2
Dcrt08	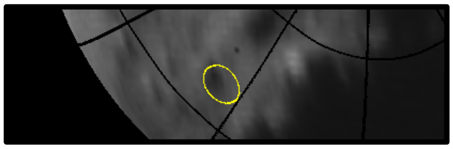	39.5S 215.4E	0.048	3
Dcrt09	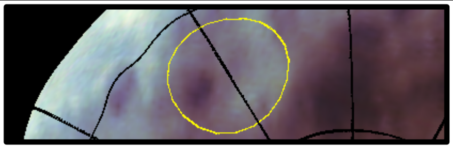	43.1S 330.2E	0.140	4

Dcrt10	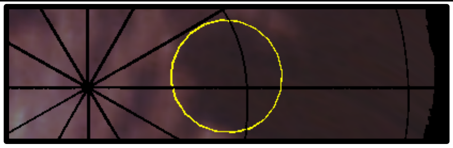	63.3S 85.3E	0.128	2
Dcrt11	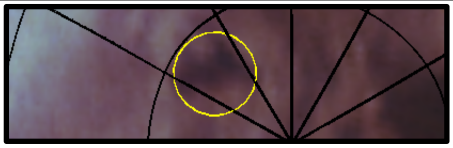	68.3S 312.9E	0.09	3
Dcrt12	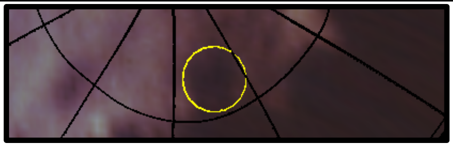	65.9S 162.4E	0.07	2
Dcrt13	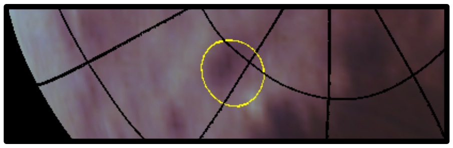	56.2S 212.9E	0.08	2
Dcrt14	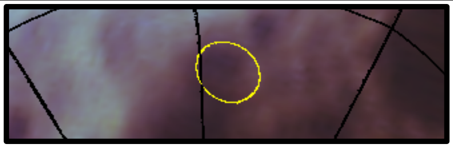	35.8S 12.9E	0.05	2
Dcrt15	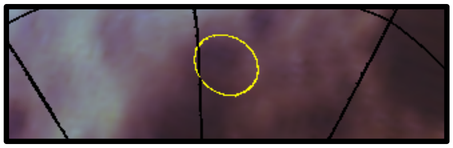	43.9S 5.3E	0.06	2
Dcrt16	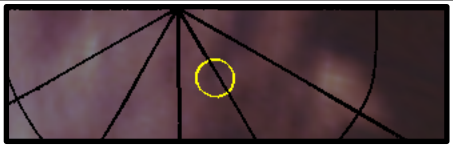	77.7S 152.6E	0.03	3

Supplementary Table 4. Candidate craters identified on Dimorphos.

**Crater Candidate**	**Image**	**Location**	**Diameter (m)**	**Depth (m)**	**Confidence**
crt02	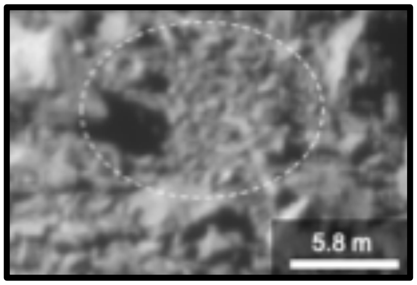	20.3N 247.7E	11.1 ± 2.1	1.9 ± 0.2	2

crt04	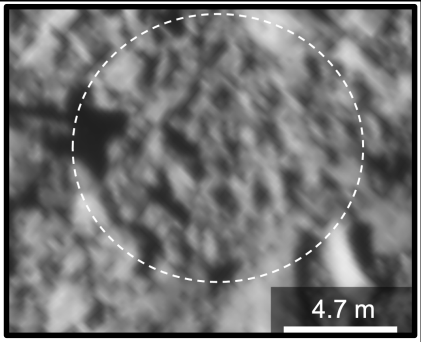	8.8N 254.2E	10.9 ± 2.1	1.4 ± 0.2	1
crt05	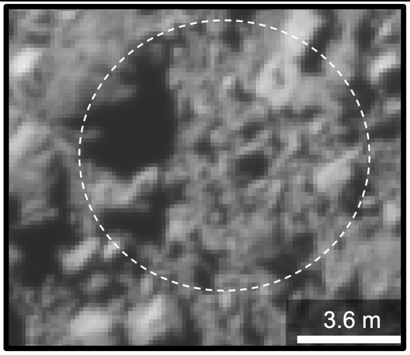	31.4S 263.3E	7.1 ± 0.7	1.3 ± 0.3	2
crt06	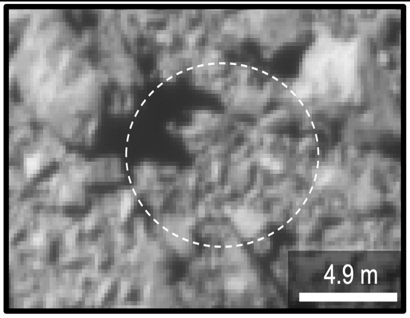	10.5S 241.6E	8.5 ± 0.9	1.3 ± 0.3	4
crt07	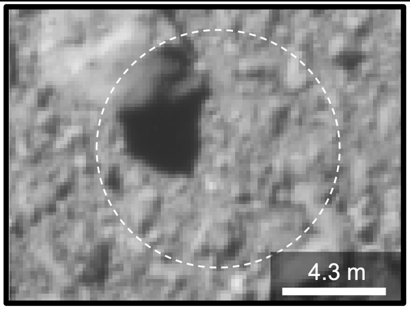	26.9S 230.8E	8.8 ± 1.1	1.6 ± 0.2	1
crt08	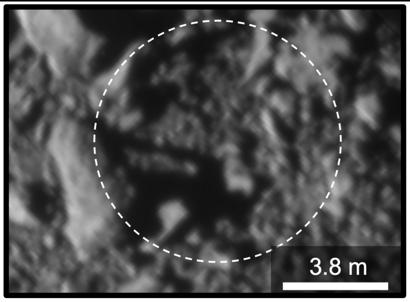	3.1N 284.7E	9.0 ± 1.4	0.99 ± 0.04	4

crt10	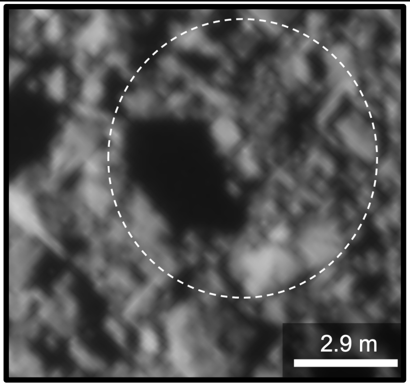	6.1S<br>283.3E	8.3 ± 0.5	0.8 ± 0.1	3
crt11	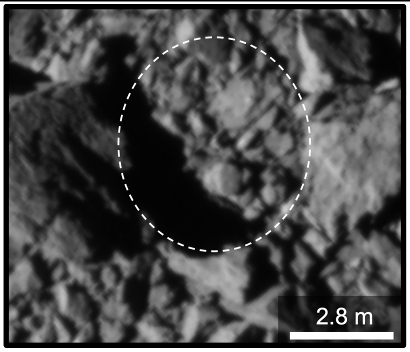	9.9S<br>266.5E	4.7 ± 0.3	0.8 ± 0.1	3
crt12	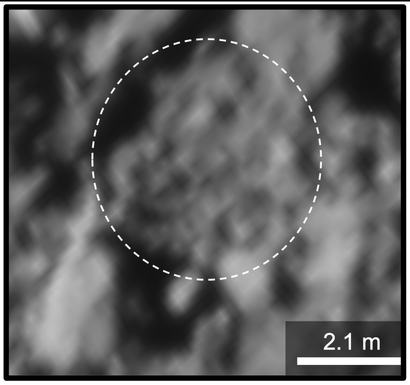	24.8S<br>271.9E	4.7 ± 0.7	0.7 ± 0.1	1
crt15	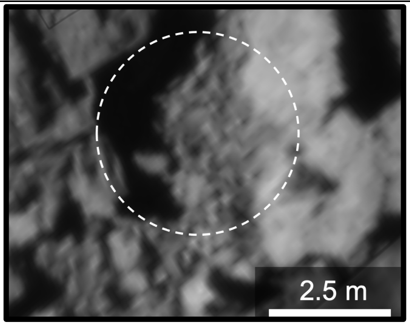	24.7<br>263.7E	5.6 ± 0.5	1.3 ± 0.2	1

crt17	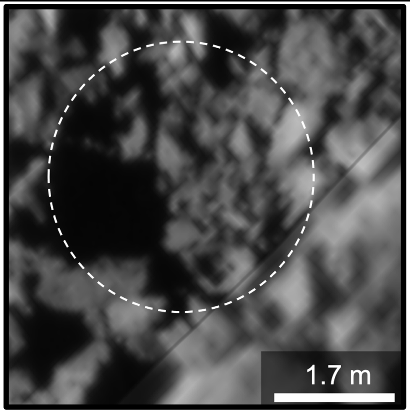	17.2S<br>275.1E	5.2 ± 0.5	0.7 ± 0.2	1
crt19	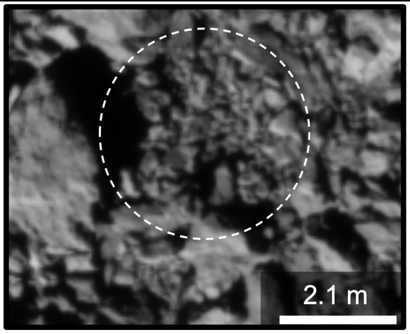	12.0S<br>271.2E	2.9 ± 0.5	0.3 ± 0.1	1
