# Supplementary material for: The geology and evolution of the Near-Earth binary asteroid system (65803) Didymos
Source: Nat Commun. 2024 Jul 30;15:6202. doi: 10.1038/s41467-024-50146-x (PMC11289119; doi:10.1038/s41467-024-50146-x)
Supplement: Supplementary file 3 — Description of Additional Supplementary Files [file 41467_2024_50146_MOESM3_ESM.pdf]

Description of Additional Supplementary Files

File Name: Supplementary Software 1
Description: The code executable provided was used to make Fig 9b of the main manuscript. See more information in the main manuscript's code availability statement.
